# Oncofertility and COVID-19—cancer does not wait

**DOI:** 10.3332/ecancer.2020.ed101

**Published:** 2020-05-08

**Authors:** Bhawna Sirohi, Tanya Buckshee Rohatgi, Matteo Lambertini

**Affiliations:** 1 Max Healthcare, Delhi, India; 2 IRCCS Ospedale Policlinico San Martino, University of Genova, Genova, Italy

**Keywords:** oncofertility, cancer, fertility, cryopreservation, COVID-19

## Abstract

The current pandemic due to the coronavirus disease 2019 (COVID-19) outbreak has forced physicians to review their current clinical practice and guidelines. Although elective procedures using assisted reproductive technologies (ART) should be preferably canceled or postponed at this time, this does not always apply to urgent procedures such as those in patients with cancer. A complete oncofertility counseling balancing the benefits and risks of undergoing fertility preservation before commencing gonadotoxic therapies (chemotherapy and/or radiotherapy) should also be provided during the COVID-19 outbreak. This article briefly highlights what patients, oncologists and fertility specialists need to keep in mind during oncofertility counseling at the time of the COVID-19 outbreak.

## Introduction

Some anticancer treatments can affect fertility [1, 2]. Oncofertility seeks to provide information to young patients with cancer on the risk of developing treatment-induced infertility and the available strategies to preserve fertility [3]. The coronavirus disease 2019 (COVID-19) pandemic has recently added additional challenges to cancer care [4–7]. This applies also to oncofertility counseling, with benefits and risks of undergoing fertility preservation before commencing gonadotoxic therapies which must be clearly addressed with each patient on an individual level. During the current COVID-19 outbreak, elective procedures using assisted reproductive technologies (ART) should be preferably canceled or postponed [8, 9]. However, this does not always apply to urgent procedures like those in young patients with cancer who are candidates to undergo gonadotoxic chemotherapy and/or radiotherapy. Urgent fertility preservation is recommended in these patients in case they wish to complete their families following the end of treatment [10, 11].

## Challenges during the pandemic

Patients face hurdles that can be as basic as getting to the tertiary hospital that offers both cancer care and fertility preservation treatments to more complex emotional thoughts dealing with the fear of both the known and unknown risks of complications from COVID-19 during cancer treatment and the additional fertility preservation treatments involving further hospital visits, potential surgery and in some units additional costs relating to COVID-19 testing and personal protective equipment (PPE) gear. Moreover, the anxieties from the fear of contracting the virus during hospital visits leading to isolation and quarantine are real enough for some to even consider opting out of fertility preserving treatments whilst fighting their most basic desire to procreate. For healthcare providers, the novel challenges of providing safe and optimal care while dealing with the undefined risks remain.

Nonetheless, where resources allow, with extra caution and strict adherence to the COVID-19 safety protocols and local guidelines, oncofertility is a feasible option, giving hope for young patients with cancer.

## The new normal: practical tips

1. Newly diagnosed eligible cancer patients should discuss before commencing treatment with their oncologist and oncofertility specialists the wish to have children to see if it is possible to safely balance this option without compromising their cancer care and further increase the risk of infections by the new severe acute respiratory syndrome coronavirus 2 (SARS-CoV-2).
2. Outline various fertility preservation procedures that can be undertaken safely with the precautions recommended by various national and international organizations including having protected pathways.
  - For male patients, sperm banking before starting anticancer treatments is standard of care.
  - For female patients, embryo/oocyte cryopreservation before starting anticancer treatments is the first option to be discussed (this can delay the start of anticancer treatment by about 2-3 weeks and requires multiple hospital visits; hence, it needs to be factored in for the urgency of starting chemotherapy or radiotherapy and the potential added risks of exposure to infections by SARS-CoV-2).
  - Ovarian tissue cryopreservation in patients that cannot wait 2-3 weeks before starting anticancer treatments can be discussed (this needs surgery, hence the pros and cons of admission and pre-surgery COVID-19 testing may be required).
  - Ovarian transposition can be considered before starting pelvic radiotherapy (this needs surgery, hence the pros and cons of admission and pre-surgery COVID-19 testing may be required).
  - Temporary ovarian suppression with luteinizing hormone-releasing hormone agonists (LHRHa) during chemotherapy is an available option to protect ovarian function during treatment (no additional hospital visits are required).
3. Detailed oncofertility counseling should clearly discuss the additional concerns during the COVID-19 outbreak whist defining all the established safety protocols in place to minimize these risks.
4. Triage patients for SARS-CoV-2 testing in accordance with local guidelines before starting any of the ART procedures.
5. Train additional members of the team (nurses, doctors, etc) on how to refill the liquid nitrogen tanks in case the lab staff is quarantined.
6. If local resource allocation allows, consider having two teams (each comprising of fertility specialist, nurse, anaesthesiologist, embryologist and witness) working by rota. An alternate team can take over in case one team comes in contact with an infected patient [9].

## Conclusion

The advances in oncofertility have given a lot of hope on preserving future fertility to cancer patients. Hence, we urge oncologists and fertility specialists to consider oncofertility counseling (which includes COVID-19-related safety concerns at this time) and work as a dedicated team to support young cancer patients optimizing their future fertility and reproductive health even during this pandemic. Let us not allow the COVID-19 outbreak to sidetrack us on this important issue.

## Conflicts of interest

Tanya Buckshee Rohatgi has no conflicts of interest. Bhawna Sirohi has received honoraria from Bayer and Roche outside the submitted work (real-world case presentations). Matteo Lambertini acted as a consultant for Roche, and received honoraria from Theramex, Takeda, Roche, and Lilly outside the submitted work.

## Funding statement

No funding was received for this work.
